# Development of Fetal Yawn Compared with Non-Yawn Mouth Openings from 24–36 Weeks Gestation

**DOI:** 10.1371/journal.pone.0050569

**Published:** 2012-11-21

**Authors:** Nadja Reissland, Brian Francis, James Mason

**Affiliations:** 1 Department of Psychology, University of Durham, Science Laboratories, Durham, United Kingdom; 2 Department of Mathematics and Statistics, Lancaster University, Lancaster, United Kingdom; 3 Wolfson Research Institute, University of Durham, Queen’s Campus, Stockton, United Kingdom; The University of Tennessee Health Science Center, United States of America

## Abstract

**Background:**

Although some research suggests that fetuses yawn, others disagree arguing that is it simple mouth opening. Furthermore there is no developmental account of fetal yawning compared with simple mouth opening. The aim of the present study was to establish in a repeated measures design the development of fetal yawning compared with simple mouth opening.

**Methodology/Findings:**

Video recordings were made of the fetal face and upper torso visualized by means of 4D full frontal or facial profile ultrasound recordings. Fifteen healthy fetuses were scanned four times at 24, 28, 32 and 36 weeks gestation. Yawning was distinguished from non-yawning in terms of the length of time it took to reach the apex of the mouth stretch, with yawns being defined as more than 50% of the total time observed. To assess changes in frequency, a Poisson mixed effects model was fitted to the count of number of yawn and simple mouth opening events with age and gender as fixed effects, and person as a random effect. For both yawns and simple mouth openings a smooth varying age effect was significant. The number of yawns observed declined with age from 28 weeks gestation, whereas simple mouth openings were less frequent and the decline was observed from 24 weeks. Gender was not significant either for yawn and simple mouth openings.

**Conclusions/Significance:**

Yawning can be reliably distinguished from other forms of mouth opening with the potential of using yawning as an index of fetal healthy development.

## Introduction

The development of yawning, a movement which has been reported in humans and many vertebrates from fetal stages to old age (e.g. [1]–[2]) remains poorly understood [3]. According to Provine [4] human are unique because in contrast to other species, for example rats, where males yawn more frequently than females [5], in humans both sexes yawn equally often. There is however, a lack of research on fetal yawning.

Research suggests that there is a U-shaped developmental progression to yawning in that premature infants yawn more frequently than term babies [6] and primary school children yawn more frequently than kindergarten children [7]. Most research on yawning, a movement defined as mouth opening with the jaw dropping, relates to the contagious nature of yawning. Provine [4] suggests that yawns are so infectious that simply thinking or reading about yawning results in a yawn in around 60% of observations [8]. Interestingly, children are immune from the contagious nature of yawning until around five years of age [9] hence not only yawning frequency but also the social context of yawning, such as contagious yawning, has a developmental component which is as yet unexplained. Contagious yawning however cannot be the reason for fetal yawning. Another theory which potentially explains fetal yawning suggests that yawning is related to central nervous system (CNS) arousal modulation and hence related to waking motility patterns [6]. This is supported by evidence that cortisol levels are increased during stress and fatigue as well as yawning [10]. However, others disagree arguing that fetuses do not yawn because they feel sleepy” [11 p: 36]. Even though the relationship between the neural network of mouth, tongue and respiratory movements is not well understood, some research suggests that the function of yawning in fetuses might lie in activity-dependent brain maturation [12].

In summary, yawning has been reported from the end of the first trimester, [1]. Sherer, Smith, & Abramowicz [13] described yawning in a case study at 20 weeks gestation. Roodenburg, Wladimiroff, van Es, & Prechtl [14] observed 9 fetuses in 2D imaging who only occasionally yawned but they were able to ascertain that jaw movements increased up to 28 weeks and then declined. Yigiter, & Kavak, [15], in a cross sectional study, report no significant changes in mouthing, yawning and sucking.

One reason for varied findings could be due to a lack of a precise definition of yawns compared with mouth opening. McMagnus, Devine, & Brandsetter [16] go so far as to argue that definitions of yawning are so varied that what has been labelled a yawn can be just a mouth opening or repeated set of mouth openings rather than yawns. Indeed, in terms of a measure of fetal neurological development [17], yawning and mouth opening are not distinguished.

Provine ([4] p532) suggests a general definition of a yawn consisting of jaws open in a wide gape, a deep inward breath followed by a shorter exhalation and a closing of the jaws. Robust differentiation between wide mouth opening and a yawn in contrast requires a dynamic definition of yawning [18]. Hence, it is essential to differentiate between wide mouth opening with the jaw dropping and yawning in terms of the timing of these mouth movements either being labelled a yawn or mouth stretch. One dynamic definition proposed by Petrikovsky, et al. [18] is a “prolonged wide opening of the mouth followed by a quicker closure of the mouth”. Petrikovsky et al. [18] used this definition of yawning in 2-D ultrasound examinations of fetal mouth movements. Applying this dynamic definition to their data they found a mean of 5 yawns in 18 of 22 healthy fetuses. In contrast, the nine anaemic fetuses studied yawned more frequently with a mean of 12 yawns observed in the 1 hour period.

The present longitudinal study examined the dynamics of yawning compared with non-yawn mouth opening in 15 healthy fetuses, 8 girls and 7 boys, observed four times over the second and third trimester of pregnancy. We expected that if yawning is a developmental process, then the frequency of yawning might change during gestation. Given previous research on human yawning we also did not expect to identify sex differences.

**Figure 1:**
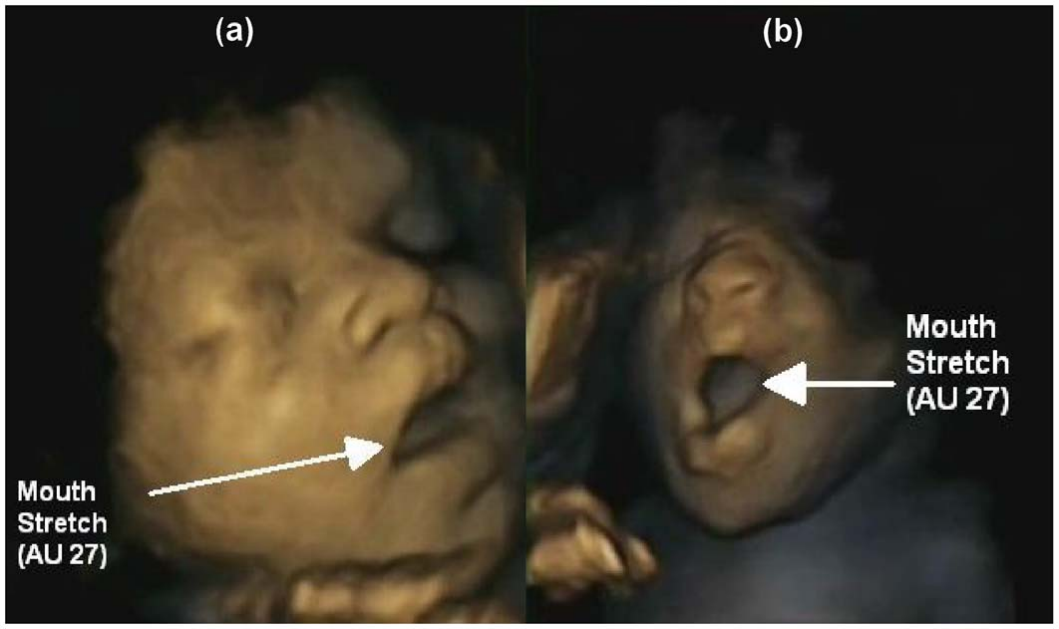
Apex of yawning and non-yawn mouth openings. The Figure demonstrates that yawning and non-yawn mouth opening cannot be distinguished from a static image at the apex of the event.

**Figure 2:**
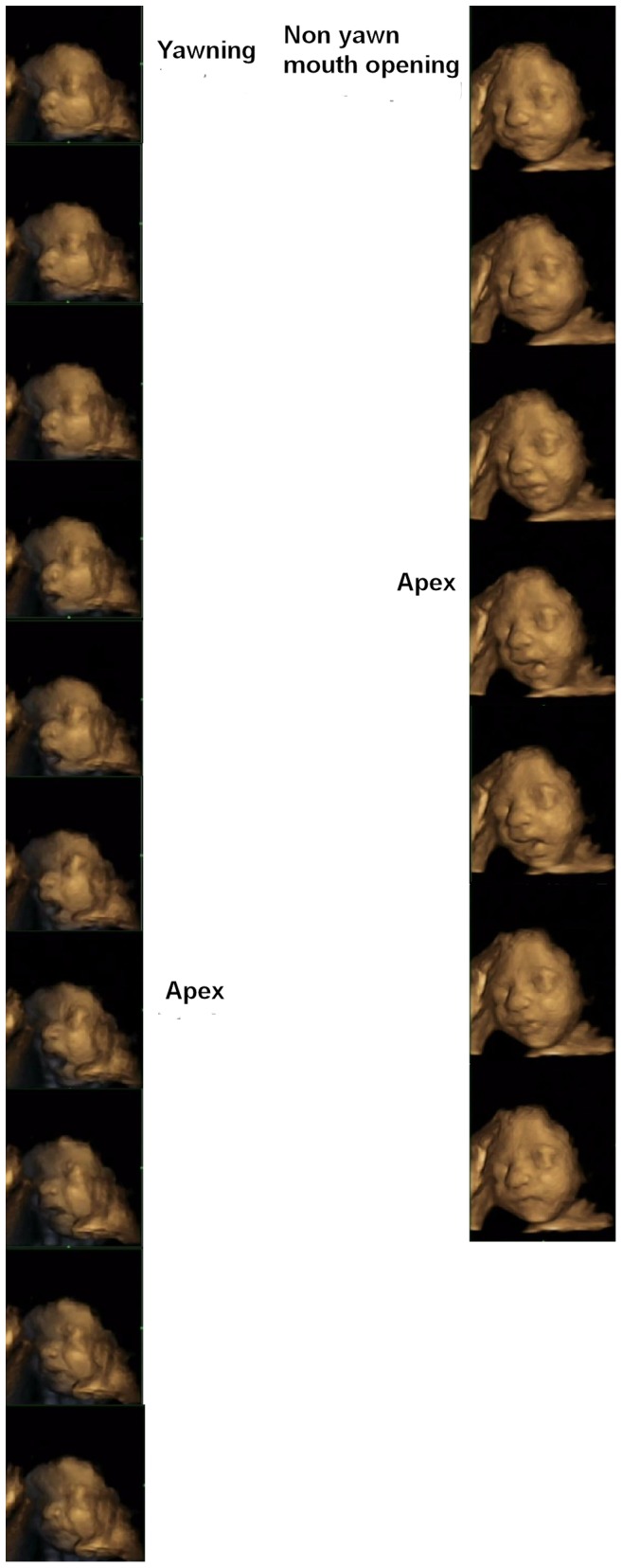
Yawning and non-yawn mouth opening sequences from mouth closed to apex and apex to mouth closed.

## Materials and Methods

### Ethics Statement

Ethical permission was granted by the County Durham and Tees Valley 2 Research Ethics Committee (REC Ref: 08/H0908/31), James Cook University Hospital. All mothers gave informed written consent.

### Participants

Fifteen healthy fetuses were scanned: 8 girls and 7 boys. The fetuses were observed four times based on their gestational age which was established at 12 weeks through measures of crown rump length and/or at the 20 week anomaly scan based on head circumference. The first scan was at a mean 24.2 weeks gestational age (range 23.5–25.0 weeks); the second at 28.0 weeks gestational age (range 27.4–28.5 weeks); the third at 32.1 gestational age (range 31.0–33.1 weeks); the fourth at 36.2 weeks gestational age (range 35.5–36.5 weeks). All participants were first time mothers with mean age 27 years (range 19–40 years), specifically recruited through the midwives of the antenatal unit of the James Cook University Hospital, Middlesbrough, UK and following ethical procedures. All fetuses were healthy, of Caucasian parents, with mean birth weight 3283 grams (ranging in weight from 2380 grams to 4160 grams). Gestational age at birth was 40 weeks (range 37–42); head circumference at birth was 34.5 cm (range 32.0 to 36.5 cm). Apgar scores resulted in a mean 9.06 at 1 minute (range 9–10) and mean 9.33 at 5 minutes (range 9–10). All new-borns were rated to be healthy by a paediatrician. One fetus born at 37 weeks and 1 day was in terms of weight at around the 10^th^ percentile and hence seemed to be at the lower limit of the normal/small range for gestational age classification [19]. We therefore also examined whether this fetus differed from the group in terms of frequency of yawning and mouth opening over gestational age.

**Figure 3:**
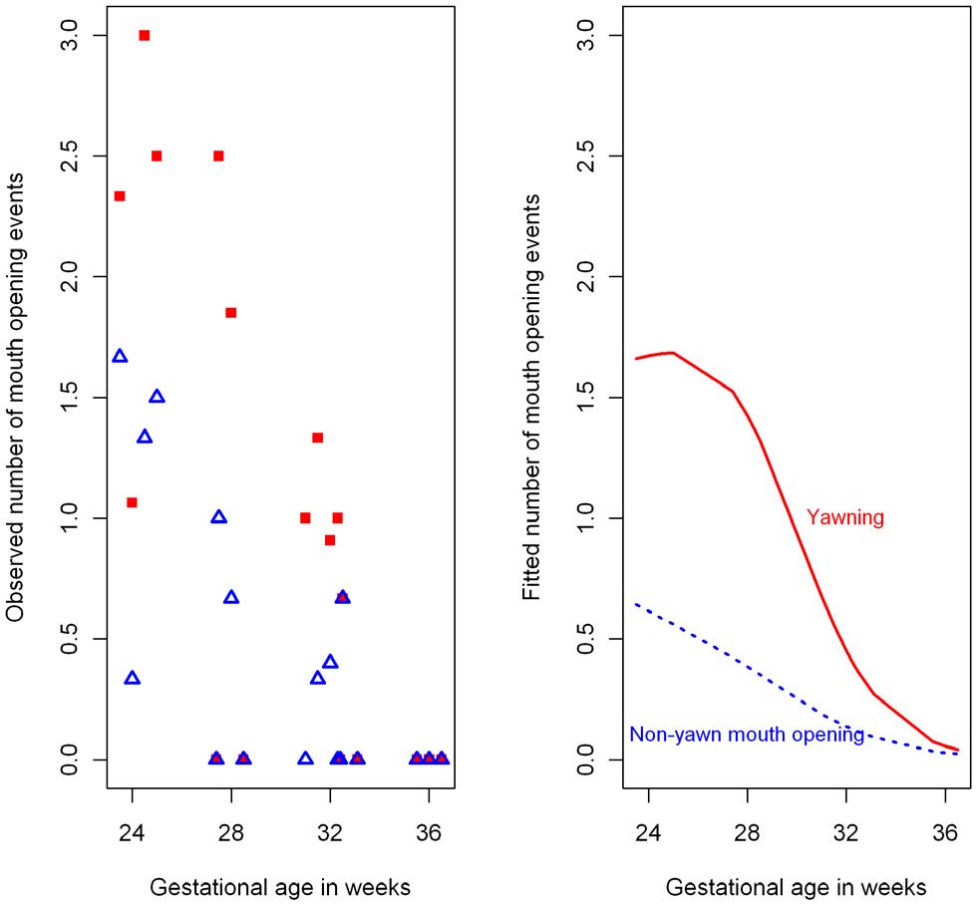
Observed and fitted yawning and non-yawn mouth opening frequencies by gestational age. Left panel: Mean observed number of yawn (red solid square) and non-yawn (blue unfilled triangle) mouth openings for each gestational age over 600 seconds of observation. Right panel: Fitted 600-second counts from Poisson mixed effects model for an average fetus for yawning (red solid line) and non-yawn (blue dashed line) mouth openings.

### Method of Data Collection

Following ethical guidelines, after mothers had completed normal 20 week anomaly scans, they were approached by the radiographer, for consent to participate in the study. Mothers who agreed received four additional scans in which fetuses were observed while active for approximately 20 minutes. During consent and again before each scan mothers were made aware that these additional scans were for research purposes and not routine medical scans and that they could withdraw from the study at any time. At the end of each scan mothers were provided with a DVD copy of their scans. The fetal face and upper torso were visualized by means of 4D full frontal or facial profile ultrasound recordings, and recorded for off line analysis with a GE Voluson 730 Expert Ultrasound System using a GE RAB4–8L Macro 4-D Convex Array Transducer. For each observation period, we coded 600 seconds of each scan (which were not necessarily consecutive) when the full face was visible; starting with the first moment when the full face could be coded. No external stimulation was applied in these observation periods. The first scan of one fetus and the last scan of a second fetus could not be coded because the complete cycle of mouth movements could not be observed during the scan. Hence the findings reflect 58 rather than 60 scans.

### Method of Coding

We distinguished between yawning and non-yawn mouth opening, based only on duration of the mouth opening. We examined all events where a mouth stretch occurred. Mouth stretch was defined by the mandible being pulled down, changing the shape of the mouth opening from oval with the long axis in the horizontal plane to one in the vertical direction, and using the objective coding system which we tested previously [20]. We measured two durations of the mouth opening event: from mouth opening start to maximum opening, and from maximum to closure. We defined a yawning event to be those mouth openings where the time to maximum opening of the mouth was of a longer duration than the time from maximum opening to closing. The remainder were labelled as non-yawn mouth openings. This definition of yawning is similar to Petrikovski et al. [18] who specified that yawning was a slow opening of the mouth (50–75% of the cycle) and faster return to the initial position (5–10% of the cycle). This cyclical behaviour was absent in non-yawn mouth openings where the dynamic of slow opening and fast closing of the mouth was absent.

Using Cohen’s Kappa, reliability was established for these scans, which were coded independently by a new coder trained in the coding system. This resulted in reliability estimates for overall reliability for all AUs coded (overall mean = .91, overall mean range.79–1.00).

Figure 1 illustrates the similarity in static images of yawn and non-yawn mouth openings. Figure 2, in contrast, shows that when 4-D dynamic scans are used, yawning and non-yawn mouth openings can be readily distinguished.

### Method of Analysis

We used a Poisson mixed effects analysis [21] to assess developmental change in the rate of yawns and non-yawn mouth openings over gestational age. This analysis models the number of events as a count variable adjusted by the length of scan as an offset, with fixed effects of age and gender, and a random individual fetus effect. The model accounts both for the skewness of the data and also allows for individual variability between fetuses in their propensity to the event. To test the effects of age, both linear age and a smooth non-linear age effects using cubic splines were fitted [22]. Testing between models was carried out using likelihood ratio tests, allowing differences in deviance to be compared to a chi-squared distribution on the appropriate degrees of freedom. The analysis uses all available scans; the two missing scans were assumed to be non-informative (that is, whether a fetal face could be observed was independent of the rate of yawning and mouth opening). The mixed effects modelling was carried out using the *glmer* function in the *lme4* package of the statistical package R. [23]; smoothing was carried out using natural B-splines with 2 degrees of freedom using the *ns* function.

## Results

Over the 58 scans, 56 yawns and 27 non-yawn mouth openings were observed in total. The rate per hour for yawns was 6.02 (s.d.9.56); whereas that for non-yawn mouth openings was 2.79 (s.d. 5.64). A Wilcoxon signed–rank test showed significant differences in these two rates (Z = 3.008; two- sided p = 0.003 on 58 df).

Given that yawning is distinguished from non-yawn mouth openings by the opening part of the movement cycle being longer compared to the closing part of the cycle, we examined the ratio of opening duration to closing duration in our sample for the 56 yawns observed. 93% of the yawns had a ratio of over 1.5 (60∶40 split), and 77% of yawns had a ratio of over 2.33 (70∶30 split).

An exploratory analysis of the 15 fetuses showed a strong decline in the mean frequency of both yawns and non-yawn mouth openings as gestational age increased. Means (with standard deviations in parentheses) for yawns at 24, 28,32 and 36 weeks were respectively 1.93 (2.17); 1.40 (2.03); 0.73 (0.96) and 0.0(0.0); means and standard deviations for the same ages for non-yawn mouth openings were lower at 1.00(1.47); 0.53(0.83); 0.33(0.72) and 0.0 (0.0) The fetus born at 37 weeks had no yawns or non-yawn mouth openings; but two other fetuses in our sample born at 40 and 41 weeks also exhibited the same behaviour. The observational data for the fetus born at 37 weeks is therefore consistent with the rest of the sample and has been retained in the statistical analysis.

### a) Yawns

To assess the changing frequency of yawns over age and gender, a Poisson mixed effects model was fitted to the count of number of yawn events with age and gender as fixed effects, and person as a random effect, to account for the repeated measures design (Figure 3).

Gender was not significant, (chi-squared = 0.109 on 1 df; p = 0.74) but linear age was significant (chi-squared = 33.42 on 1 df; p<0.001), with the counts declining with age.

A non-linear age effect was fitted using a natural cubic spline with two degrees of freedom, and the non-linear model showed a significant improvement over the linear model (chi-squared = 7.01 on 1 df; p<0.01).

### b) Non-yawn Mouth Openings

The same model was fitted to the number of non-yawn mouth opening events (Figure 3).

Gender was again not significant, (chi-squared = 0.584 on 1 df; p = 0.44) but linear age was significant (chi-squared = 17.18 on 1 df; p<0.001), with the counts again declining with age.

A non-linear age effect was fitted using a natural cubic spline with two degrees of freedom, but in this case there was no significant improvement over the linear model (chi-squared = 1.13 on 1 df; p = 0.29).

### c) Comparison between Yawn and Non Yawn Mouth Openings

A combined generalised linear mixed model was fitted which allowed a comparison of the declining trajectories for yawn and non- yawn frequency rates over age. Three models were fitted, a model (Model 1) with non-linear age only, a model (Model 2) with non-linear age and an indicator variable representing the type of mouth opening (yawning = 1, non-yawning = 0), and a model fitting different intercepts and non-linear age functions to each type of mouth opening (Model 3). Model 2 showed a significant improvement over Model 1 (Chi-squared = 12.20 on 1 df, p<0.001) but Model 3 showed no significant improvement over Model 2. (Chi-squared = 0.43 on 2 df, p = 0.805). The estimate of the indicator variable effect in model 2 was 0.782 and the exponential of this number exp(0.782 = 2.19) can be interpreted as a multiplicative effect for yawning rate over the non-yawn effect, with both having the same trajectory shape over age, and with yawning rate just over double the non-yawning rate.

## Discussion and Conclusion

There are a number of hypotheses explaining the ubiquitous behaviour of yawning [24]. One of them is that yawning is a response to elevated levels of carbon dioxide or depressed levels of oxygen in the blood which has been shown to be a false association [2]. The results of this study demonstrate that yawning can be observed in healthy fetuses and replicates previous studies with 2-D images. In contrast to previous research we could also show that although healthy fetuses vary in the frequency of yawns observed overall, the repeated measures design allowing an observation of the same fetuses at 24 to 36 weeks gestation in 4 weekly intervals showed that in healthy fetuses the frequency of yawning declines over time. Specifically, in our longitudinal study following 15 fetuses, we observed yawns when the fetus was active at 24 weeks but not in the similarly active fetus at 36 weeks (Figure 3). This finding corroborates work by Giganti et al. [6] who videotaped 12 healthy infants ranging in age from 31 to 40 weeks (post gestational age) and recorded 1.10+−0.7 yawns for the infants in a 24 hour period which decreased with age to zero yawns.

Hence, this research could support the suggestions that yawning is related to CNS maturation e.g. [25]–[26] rather than arousal modulation. Furthermore, supporting other results on similar yawning frequency in human males and females, in our research we did not find any sex differences in yawning frequency.

In summary, the importance and function of yawning is still unclear. Some researchers found an association of yawning with neurological functioning e.g. [25]–[27]; others found a relationship between yawning and Parkinson’s disease [28]. Yet other research argued that yawning has a thermo-regulative function [29]. A further hypothesis states that blood cortisol levels might be the cause for yawning [30] or that yawning could be due to the increase of venous blood to the heart of the fetus [18]. A developmental function of yawning could be related to jaw movements, which are important in the prenatal and postnatal development of the cartilage in the temporo-mandibular joint, enabling normal mouth movements [31]. In contrast to these medical reasons for yawning others argue that yawning has a communicative function [32]. In our sample, we can exclude the communicative function of yawning. However, other hypotheses could be supported. Given that the frequency of yawning in our sample of healthy fetuses declined from 24 to 36 weeks gestation, it is possible that yawning and simple mouth opening have a maturational function early in gestation. Although, yawning and simple mouth opening have the same trajectory shape over age it is notable that the yawning rate is just over double the non-yawning rate. In order to exclude the potential function of cortisol in yawning, in future research it would be important to measure maternal cortisol levels at the time of observing fetal yawns.

## Acknowledgments

We thank the mothers who took part in the study, Kendra Exley who performed the 4-D scans, Dr. Karen Lincoln for her support, as well as the independent coders of fetal facial movements.
